# Mpox in the Emergency Department: A Case Series

**DOI:** 10.5811/cpcem.1259

**Published:** 2023-10-27

**Authors:** Michael Musharbash, Madeline DiLorenzo, Nicholas Genes, Vikramjit Mukherjee, Amanda Klinger

**Affiliations:** * New York University School of Medicine, Department of Emergency Medicine, New York; † New York University School of Medicine, Department of Internal Medicine, New York

**Keywords:** *mpox*, *monkeypox*, *case series*, *operations*, *pandemic response*

## Abstract

**Introduction:**

We sought to describe the demographic characteristics, clinical features, and outcomes of a cohort of patients who presented to our emergency departments with mpox (formerly known as monkeypox) infection between May 1–August 1, 2022.

**Case Series:**

We identified 145 patients tested for mpox, of whom 79 were positive. All positive cases were among cisgender men, and the majority (92%) were among men who have sex with men. A large number of patients (39%) were human immunodeficiency virus (HIV) positive. There was wide variation in emergency department (ED) length of stay (range 2–16 hours, median 4 hours) and test turnaround time (range 1–11 days, median 4 days). Most patients (95%) were discharged, although a substantial proportion (22%) had a return visit within 30 days, and 28% ultimately received tecrovirimat.

**Conclusion:**

Patients who presented to our ED with mpox had similar demographic characteristics and clinical features as those described in other clinical settings during the 2022 outbreak. While there were operational challenges to the evaluation and management of these patients, demonstrated by variable lengths of stay and frequent return visits, most were able to be discharged.

CPC-EM Capsule What do we already know about this clinical entity?

*Mpox is a zoonotic disease endemic to parts of Africa. In 2022, an outbreak of mpox occurred internationally.*

What makes this presentation of disease reportable?

*This case series highlights the demographic and clinical characteristics of 79 mpox cases in an urban emergency department in the United States.*

What is the major learning point?

*Patients with mpox in this case series were primarily men who have sex with men; most cases were managed in the outpatient setting with the help of telemedicine.*

How might this improve emergency medicine practice?

*Response to future infectious disease outbreaks might benefit from establishing protocols to evaluate, manage, and follow up with patients.*

## INTRODUCTION

Mpox is a viral illness endemic to parts of West and Central Africa that causes fever, lymphadenopathy, and a rash that typically evolves over the course of weeks.^1^ Transmission often occurs via contact with an infected animal, although human-to-human transmission has been documented through several mechanisms.^1^ Structurally, the mpox virus is closely related to variola virus, which causes smallpox.^2^ Vaccination with live (ACAM2000) or attenuated (JYNNEOS) vaccinia virus, which are also used to protect against smallpox, is thought to confer protection against mpox.^2^ Management of mpox is mainly supportive, although tecovirimat (TPOXX) is an emerging antiviral treatment.^2^

In 2022, an outbreak of mpox occurred internationally among patients who had never traveled to endemic regions.^1^ The first cases of mpox in the United States were noted in May 2022, with average daily case counts reaching a peak of approximately 450 nationally and 70 in New York City (NYC) in early August 2022, and have since declined.^3^ The reasons for this decline remain to be fully elucidated but may be due in part to vaccination efforts^4^ and behavioral changes within at-risk populations.^5^

During the early months of the outbreak, there were anecdotal reports of clinicians turning away patients with suspected mpox.^6^ Without alternative options, emergency departments (ED) became one of the primary sites of care for patients with suspected mpox.^7^ Here, we describe a case series of all patients who tested positive for mpox between May 1–August 1, 2022 at two urban, high-volume EDs with annual visits of greater than 100,000 patients per year. We created a deidentified, structured, case-series spreadsheet based on variables of interest (Appendix). These variables were derived from previous studies and our clinical experience.^1^ We then reviewed patient charts retrospectively and entered data into this spreadsheet. Descriptive statistics were derived from this review.

## CASE SERIES

During this period, 145 patients were identified as persons under investigation (PUI) and tested for mpox. All samples were collected in the ED and submitted to the NYC Public Health Laboratory. There were 94 PUIs at the Bellevue Hospital Center (BHC) ED and 61 at the New York University Langone Medical Center (NYULMC) ED. A total of 79 (54%) PUIs had a positive mpox test, 46 (32%) had a negative mpox test, and 20 (14%) had missing or inconclusive tests. Demographic characteristics of positive mpox cases are described in Table 1.

**Table 1.** Demographic characteristics of mpox cases.

Measure	Mpox cases (N = 79)
Median age (range, SD) – years	34 (21–61, 8. 9)
Gender – no. (%)	
Male	79 (100%)
Female	0 (0%)
Transgender	0 (0%)
Race – no. (%)	
White	34 (43%)
Black	17 (22%)
Asian	2 (3%)
Native American or Pacific Islander	1 (1%)
Other	20 (25%)
Unknown	5 (6%)
Ethnicity – no. (%)	
Hispanic	23 (29%)
Non-Hispanic	55 (70%)
Unknown	1 (1%)
Sexual orientation – no. (%)	
MSM	73 (92%)
Non-MSM	3 (4%)
Unknown	3 (4%)
HIV status – no. (%)	
Positive	30 (38%)
Negative	40 (51%)
Unknown	9 (11%)
Mpox vaccine status – no. (%)	
Vaccinated	11 (14%)
Unvaccinated	36 (46%)
Unknown	32 (41%)

*No*, number; *MSM*, men who have sex with men.

All mpox cases in our series were among cisgender men, 92% of which were among men who have sex with men (MSM). In 30 (39%) of the mpox cases, patients reported being human immunodeficiency virus (HIV) positive. Of these cases, 19 were well controlled (defined as a cluster of differentiation 4 [CD4] count greater than 200 cells per microliter (μL) and an undetectable HIV RNA; three were poorly controlled (defined as a CD4 count less than 200 cells/μL and/or a detectable HIV RNA); and eight did not have a recent CD4 count documented. Of mpox cases reported, 14% received at least one dose of the JYNNEOS vaccine. Of note, JYNNEOS vaccination for high-risk individuals without known exposure to mpox did not become available in NYC until late June 2022.

Demographic characteristics of positive mpox cases are described in Table 2. Skin lesions were noted in all mpox cases. Of the positive cases, 72% were found to have genital lesions, and 77% were found to have at least one systemic symptom. Systemic symptoms included fever (58%), chills (22%), lymphadenopathy (35%), myalgias (33%), and sore throat (13%). The most common complications were proctitis (23%), rectal bleeding (10%), cellulitis (10%), phimosis (6%), and eyelid involvement (3%).

**Table 2.** Clinical characteristics of mpox cases.

Measure	Mpox cases (N = 79)
Skin lesions – no. (%)	79 (100%)
Non-genital lesions	63 (80%)
Genital lesions	57 (72%)
Penile lesions	38 (48%)
Rectal lesions	25 (32%)
Oral lesions	12 (15%)
Systemic symptoms – no. (%)	61 (77%)
Fever	46 (58%)
Chills	17 (22%)
Lymphadenopathy	28 (35%)
Myalgias	26 (33%)
Sore throat	10 (13%)
Complications – no. (%)	
Penile edema/phimosis	5 (6%)
Rectal pain/proctitis	18 (23%)
Rectal bleeding	8 (10%)
Bacterial superinfection	8 (10%)
Ocular involvement (eyelid)^*^	2 (3%)
Chlamydia co-infection	
Tested – no. (%)	23 (29%)
Positive – no. (% tested)	2 (9%)
Gonorrhea co-infection	
Tested – no. (%)	23 (29%)
Positive – no. (% tested)	3 (13%)
Syphilis co-infection	
Tested – no. (%)	26 (33%)
Positive – no. (% tested)	3 (12%)
Herpes simplex virus co-infection	
Tested – no. (%)	14 (18%)
Positive – no. (% tested)	0 (0%)
HIV co-infection	
Tested – no. (% without known HIV-positive status)	24 (49%)
Positive – no. (% without known HIV-positive status tested)	1 (4%)

* None of these patients had intraocular lesions or vision changes noted.

*No*, number; *HIV*, human immunodeficiency virus.

Management and outcomes of positive mpox cases are described in Table 3. In addition to symptomatic management, some mpox cases were empirically treated for gonorrhea and chlamydia (16%), syphilis (6%), herpes simplex virus (HSV) (4%), bacterial pharyngitis (6%), and cellulitis (10%). Among patients with mpox who were tested for co-infection with sexually transmitted infections (STI), 9% had chlamydia, 13% had gonorrhea, and 12% had syphilis. None who were tested for herpes simplex virus were found to be positive. There was one new HIV diagnosis.

**Table 3.** Management and outcomes of mpox cases.

Measure	Mpox cases (N = 79)
Empiric treatment	
Gonorrhea and chlamydia – no. (%)	13 (16%)
Syphilis – no. (%)	5 (6%)
Herpes simplex virus – no. (%)	3 (4%)
Strep pharyngitis – no. (%)	5 (6%)
Cellulitis – no. (%)	8 (10%)
Disposition – no. (%)	
Discharged	75 (95%)
Admitted	4 (5%)
Median inpatient admission length of stay (range, SD) – days	4.5 (4–9, 2.4)
Median emergency department length of stay (range, SD) – hours†	4 (2–16, 3.0)
Median test turnaround time (range, SD) – days	4 (1–11.2)
TPOXX prescribed – no. (%)	22 (28%)
Revisits related to mpox within 30 days – no. (%)	17 (22%)

† For patients with multiple visits, length of stay was determined from the emergency department visit during which they were tested.

*No*, number; *SD*, standard deviation; *TPOXX*, tecovirimat.

Among mpox cases, the median length of stay (LOS) in the ED was four hours, and 95% were discharged from the ED. Among the four who were admitted, three were HIV positive, and two had CD4 counts less than 200 cells/μL. The reasons for admission included inability to isolate, psychiatric illness requiring admission, and severe mpox symptoms. Median length of hospital admission was 4.5 days. Twenty-two percent of mpox cases had a return visit to our ED for a reason related to mpox infection. The median time from ED visit to receiving a test result was four days. Ultimately, 28% received TPOXX from our institutions.

## DISCUSSION

The recent mpox epidemic disproportionally affected men, MSM, and people who live with HIV.^1^ Patients with mpox in the recent outbreak often had co-infection with STIs.^1^ This data has been reflected in reports from the United Kingdom,^8^ Spain,^9^ and the US.^10^ Data from our case series is consistent with those of previous studies but is unique in its focus on the ED as the site of care.

We noted a wide range in the ED LOS. In our clinical experience, one major contributor to LOS was obtaining approval for mpox testing from the NYC Public Health Laboratory. During most of the period of this case series, this was the only way to obtain a test. The process could take anywhere from a few minutes up to several hours. Other potential contributors to LOS included the following: variable familiarity of hospital staff with the mpox testing process leading to delays in collecting the test; stigma associated with mpox resulting in reticence of staff members to enter rooms; and the time required to prepare and terminally clean each isolation room. Additionally, at BHC there was a special pathogens team that was consulted to evaluate and perform testing of patients under investigation (PUIs). The addition of a consulting service may have contributed to ED LOS.

We also noted that more than one in five mpox cases had a second visit to the ED during the study period. Reasons for return visits included worsening symptoms, not having been tested for mpox during their first visit, request for TPOXX prescription, and work clearance. Although some revisits could potentially have been prevented by better recognition and management of symptoms during the initial visit, others were the natural consequence of the ED being the primary site of care for these patients. Although the ED is often a patient’s primary site of care, return visits could potentially be avoided by improvements in arranging follow-up care.

Admissions were rare and tended to occur in patients with complex medical, psychiatric, and social histories. The majority of patients could potentially have been managed outside the ED, underscoring the lack of sufficient outpatient services for people with mpox during this time. This was true in both public and private healthcare environments. Ultimately, 28% of mpox cases received TPOXX. We attribute this success to institutional protocols at each of our sites, which involved designating a member of the special pathogens team (at BHC) or ED follow-up center (at NYULMC) to arrange follow up with PUIs who tested positive for mpox. The majority of these follow-up visits occurred via telehealth, underscoring the importance of creating a multidisciplinary team across various clinical environments for managing public health emergencies.

## LIMITATIONS

While our findings suggest a pattern of demographic and clinical characteristics that should raise suspicion for mpox infection, we were unable to demonstrate statistical correlation using our study design. Additionally, while our data is consistent with that of other studies from the 2022 outbreak, the current epidemiological landscape may be different, particularly with the widespread vaccination effort among at-risk populations. Finally, although our study did take place at multiple sites, it suffered from local bias and may not be reflective of the experience outside NYC.

## CONCLUSION

Overall, our findings demonstrate that patients who presented to the ED with mpox infection were similar to those who presented in other clinical settings with regard to demographics, clinical features, and co-infections. While there were significant operational challenges to the management of these patients in the ED—demonstrated by variable lengths of stay and frequent return visits—potential solutions were identified along the way, most notably the use of telemedicine to arrange follow up. Most patients were ultimately able to be discharged.

## Supplementary Information



## References

[1] Thornhill JP Barkati S Walmsley S et al. Monkeypox virus infection in humans across 16 countries — April–June 2022. N Engl J Med. 2022;387(8):679–91. 35866746 10.1056/NEJMoa2207323

[2] Xiang Y White A. Monkeypox virus emerges from the shadow of its more infamous cousin: family biology matters. Emerg Microbes Infect. 2022;11(1):1768–77. 35751396 10.1080/22221751.2022.2095309 PMC9278444

[3] Kava CM Rohraff DM Wallace B et al. Epidemiologic features of the monkeypox outbreak and the public health response — United States, May 17–October 6, 2022. Morb Mortal Wkly Rep. 2022;71(45):1449–56. 10.15585/mmwr.mm7145a4 PMC9707350 36355615

[4] Kriss JL Boersma PM Martin E et al. Receipt of first and second doses of JYNNEOS vaccine for prevention of monkeypox — United States, May 22–October 10, 2022. Morb Mortal Wkly Rep. 2022;71(43):1374–8. 10.15585/mmwr.mm7143e2 PMC9620573 36301741

[5] Delaney KP Sanchez T Hannah M et al. Strategies adopted by gay, bisexual, and other men who have sex with men to prevent monkeypox virus transmission — United States, August 2022. Morb Mortal Wkly Rep. 2022;71(35):1126–30. 10.15585/mmwr.mm7135e1 PMC9472779 36048582

[6] Vasan A. New York City Department of Health and Mental Hygiene Commissioner Letter: Providing care for patients with monkeypox. Published online July 18, 2022. Available at : https://www.nyc.gov/assets/doh/downloads/pdf/monkeypox/letter-providing-care-for-patients.pdf. Accessed November 24, 2022.

[7] Gonsalves GS Mayer K Beyrer C. Déjà vu all over again? Emergent monkeypox, delayed responses, and stigmatized populations. J Urban Health. 2022;99(4):603–6. 35916973 10.1007/s11524-022-00671-1 PMC9345008

[8] Patel A Bilinska J Tam JCH et al. Clinical features and novel presentations of human monkeypox in a central London centre during the 2022 outbreak: descriptive case series. BMJ. Published online 2022:e072410. 35902115 10.1136/bmj-2022-072410 PMC9331915

[9] Iñigo Martínez J Gil Montalbán E Jiménez Bueno S et al. Monkeypox outbreak predominantly affecting men who have sex with men, Madrid, Spain, 26 April to 16 June 2022. Eurosurveillance. 2022;27(27). 10.2807/1560-7917.ES.2022.27.27.2200471 PMC9264731 35801519

[10] Philpott D Hughes CM Alroy KA et al. Epidemiologic and clinical characteristics of monkeypox cases — United States, May 17–July 22, 2022. Morb Mortal Wkly Rep. 2022;71(32):1018–22. 10.15585/mmwr.mm7132e3 PMC9400536 35951487

