# Supplementary material for: Mpox in the Emergency Department: A Case Series
Source: Clin Pract Cases Emerg Med. 2023 Oct 27;7(4):210–4. doi: 10.5811/cpcem.1259 (PMC10855293; doi:10.5811/cpcem.1259)
Supplement: Supplementary file 1 [file cpcem-7-210-s001.docx]

**Appendix: Structured Case Series Spreadsheet (Column Headings)**

Demographics

- Age
- Sex
- Race
- Ethnicity
- Sexual Orientation
- Human Immunodeficiency Virus Status (pre-testing)
- Cluster of Differentiation 4 count
- Monkeypox Vaccination Status

Clinical Features

- Systemic Symptoms
  - Fever
  - Chills
  - Lymphadenopathy
  - Myalgias
  - Sore throat
- Non-Genital Rash
  - Genital Rash
  - Penile lesions
  - Rectal lesions
  - Vaginal lesions
  - Oral lesions
- Complications
  - Penile edema/phimosis
  - Rectal pain/proctitis
  - Rectal bleeding
  - Bacterial superinfection

Co-infections (gonorrhea, chlamydia, syphilis, herpes simplex virus, or not tested)

Outcomes

- Emergency department length of stay (if discharged)
- Hospital length of stay (if admitted)
- Test result time
- Empiric treatment
- Disposition
- Return Visits
- Tecovirimat given
